# Circadian rhythm disruption upregulating *Per1* in mandibular condylar chondrocytes mediating temporomandibular joint osteoarthritis via GSK3β/β-CATENIN pathway

**DOI:** 10.1186/s12967-024-05475-2

**Published:** 2024-07-15

**Authors:** Jiaming Wei, Yuxuan Wang, Shaoqin Tu, Sai Zhang, Yi Feng, Yuluan Hou, Hong Ai, Zheng Chen

**Affiliations:** 1 Department of Stomatology, The Third Affiliated Hospital of Sun Yat-sen University, 600 Tianhe Road, Guangzhou, 500630 China; 2 Department of Stomatology, Shenzhen Sixth People’s Hospital (Nanshan Hospital), Huazhong University of Science and Technology Union Shenzhen Hospital, Shenzhen, China

**Keywords:** Temporomandibular joint osteoarthritis, Circadian rhythm, Clock gene *Per1*, Mandibular condylar chondrocytes, GSK3β/β-CATENIN pathway

## Abstract

**Background:**

Temporomandibular joint osteoarthritis (TMJOA) has a high incidence rate, but its pathogenesis remains unclear. Circadian rhythm is an important oscillation in the human body and influences various biological activities. However, it is still unclear whether circadian rhythm affects the onset and development of TMJOA.

**Methods:**

We disrupted the normal rhythm of rats and examined the expression of core clock genes in the mandibular condylar cartilage of the jaw and histological changes in condyles. After isolating rat mandibular condylar chondrocytes, we upregulated or downregulated the clock gene *Per1*, examined the expression of cartilage matrix-degrading enzymes, tested the activation of the GSK3β/β-CATENIN pathway and verified it using agonists and inhibitors. Finally, after downregulating the expression of *Per1* in the mandibular condylar cartilage of rats with jet lag, we examined the expression of cartilage matrix-degrading enzymes and histological changes in condyles.

**Results:**

Jet lag led to TMJOA-like lesions in the rat mandibular condyles, and the expression of the clock gene *Per1* and cartilage matrix-degrading enzymes increased in the condylar cartilage of rats. When *Per1* was downregulated or upregulated in mandibular condylar chondrocytes, the GSK3β/β-CATENIN pathway was inhibited or activated, and the expression of cartilage matrix-degrading enzymes decreased or increased, which can be rescued by activator and inhibitor of the GSK3β/β-CATENIN pathway. Moreover, after down-regulation of *Per1* in mandibular condylar cartilage in vivo, significant alleviation of cartilage degradation, cartilage loss, subchondral bone loss induced by jet lag, and inhibition of the GSK3β/β-CATENIN signaling pathway were observed. Circadian rhythm disruption can lead to TMJOA. The clock gene *Per1* can promote the occurrence of TMJOA by activating the GSK3β/β-CATENIN pathway and promoting the expression of cartilage matrix-degrading enzymes. The clock gene *Per1* is a target for the prevention and treatment of TMJOA.

## Introduction

Temporomandibular joint disorder (TMD) is one of the commonest oral diseases, characterized by facial muscle pain, temporomandibular joint clicking, and jaw movement disorders, whose prevalence rate in adults can reach up to 31% [1]. Temporomandibular joint osteoarthritis (TMJOA) is a common type of TMD, often involving bony destruction of the condyle and joint fossa, along with cyst or osteophyte formation [2–4]. TMJOA can lead to jaw movement disorders, limited mouth opening, and severe condylar resorption, which can result in mandibular retrusion or mandibular deviation, impact the patient’s facial appearance and affect both their physical and psychological well-being [5]. The etiology of TMJOA remains unclear, and many studies have confirmed that its occurrence is influenced by various factors such as age, gender, autoimmune diseases, hormones, trauma, occlusion, etc. [6, 7]. Mandibular condylar chondrocytes are the only cells present in the temporomandibular joint cartilage, and disruption of normal cartilage metabolism in condylar chondrocytes due to external or internal factors can lead to the onset and development of TMJOA [8–10].

Circadian rhythm is a continuous biological rhythm that exists within organisms, with a cycle of about 24 h, which is an oscillation of periodic physiological activities within the body. The circadian rhythm regulates important life activities such as body temperature, hormone secretion, sleep–wake cycles, etc. [11, 12]. The circadian rhythm is found to be controlled by clock genes. Many core clock genes, including *Clock*, *Bmal1*, *Cryptochrome 1*(*Cry1*), *Cryptochrome 2*(*Cry2*), *Period 1*(*Per1*), *Period 2*(*Per2*), etc. have been discovered and act an important role in various life activities. These genes regulate the circadian rhythm in the central and peripheral systems of organisms [13, 14]. Similarly, peripheral rhythms also exist in cartilage tissue. Cartilage metabolism itself undergoes rhythmic changes [15–17]. Clock genes also regulate cartilage metabolism and influence the occurrence and progression of osteoarthritis. Studies have shown that patients with knee osteoarthritis exhibited lower expression of the clock genes NR1D1 and BMAL1 in knee joint cartilage compared to normal individuals [18]. Conditional knockout of *Bmal1* in mouse cartilage tissue disrupted the rhythmic oscillation in chondrocytes, decreased the expression of cartilage metabolism related genes including *Sox9*, *Acan*, and *Col2a1*, leading to degenerative changes in mouse articular cartilage [19].

A few reports suggest that the clock genes are potentially correlated to the TMJOA. For examples, in a study using unilateral occlusal trauma in rats to induce TMJOA, RNA sequencing analysis of the condylar cartilage revealed the differential expression of *Per2* compared to control group, indicating that *Per2* is a potential therapeutic target gene for TMJOA [20]. Another study used chronic sleep deprivation to disrupt the normal sleep timing in Wistar rats and found that the condyles of the temporomandibular joint exhibited osteoarthritis-like changes such as osteophyte formation. The study also showed that the expression of the clock gene *Bmal1* was decreased and demonstrated that *Bmal1* in mandibular condylar chondrocytes could regulate the degradation of cartilage matrix, thus contributing to the pathological process of TMJOA [21].

These studies reveal that circadian rhythm could act a key role in TMJOA, while up to now, there is currently no research substantially investigate their correlations. The aim of this study is to establish a jet lag rat model to explore the effects of circadian rhythm disruptions on TMJOA, and to investigate the specific molecular mechanisms through in vivo and in vitro experiments regarding the impact of clock genes on TMJOA.

## Materials and methods

### Construction of jet lag rat model and expression of core clock genes within 24 h

**Fig. 1:**
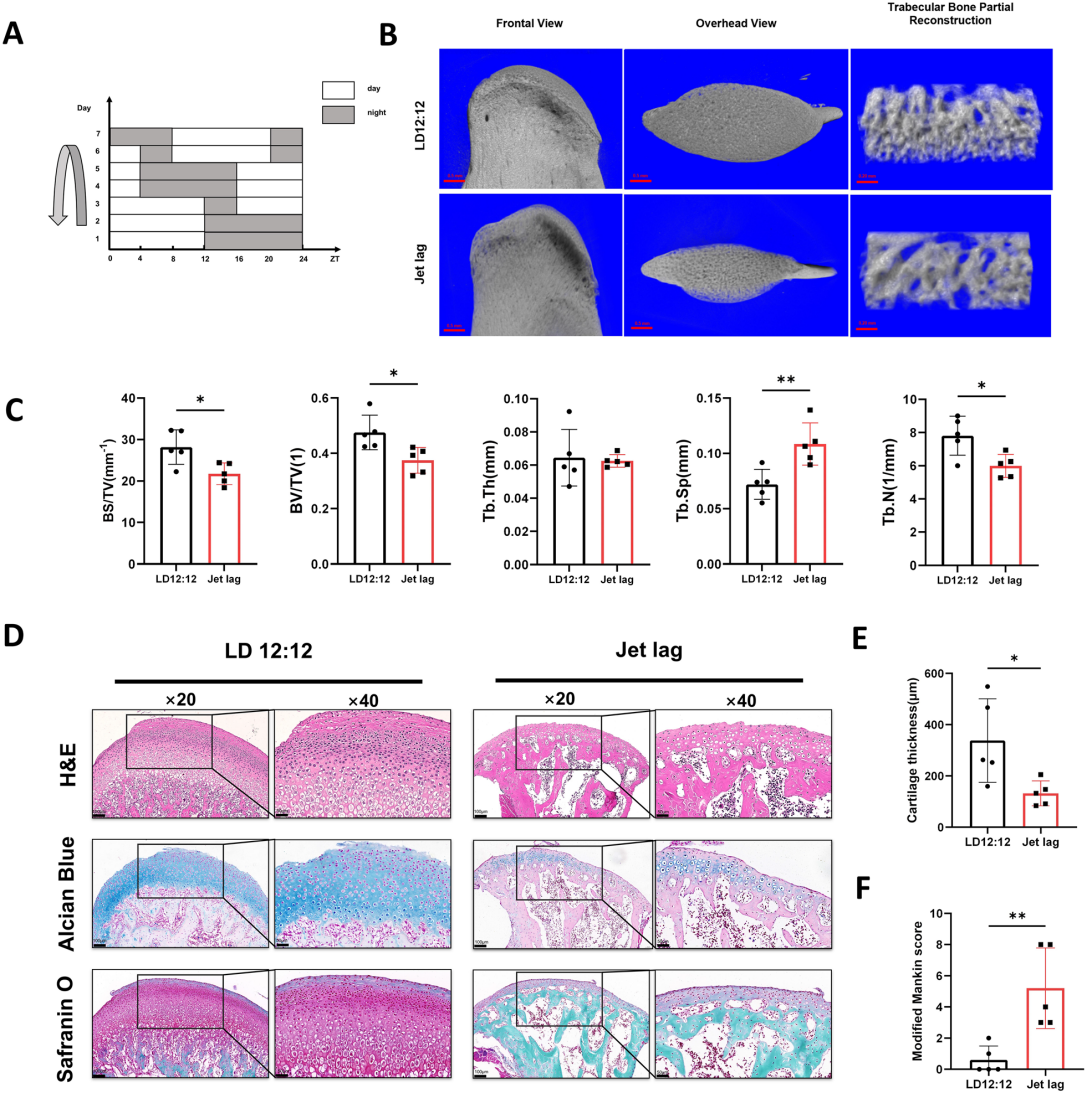
Chronic circadian rhythm disruption leads to TMJOA-like lesions in the rat mandibular condyles.** A** Light/dark timing schematic diagram of chronic progressive jet lag. **B** Three-dimensional reconstruction images of condyles from LD12:12 and Jet lag groups by Micro-CT. **C** Quantitative evaluation of Micro-CT data including BS/TV, BV/TV, Tb.Th, Tb.Sp and Tb.N. **D** Representative sections of H&E, Alcian Blue-Nuclear Fast Red and Safranin O-Fast Green staining of two groups. **E** Cartilage thickness of two groups. **F** Modified Mankin scores of two groups. n = 5, *P < 0.05. **P < 0.01

100 male Sprague Dawley rats (6w, 150-160 g. Multiple studies have shown that estrogen affects the occurrence and development of TMJOA [22–25]. To eliminate the interference of estrogen, all the rats used were male) were randomly divided into Jet lag group and LD12:12 group, with 50 rats in each group. All operations are carried out in accordance with animal ethics (approved by Ethics Committee of Sun Yat-sen University, China, protocol No. 2021001661). Prior to the start of the experiment, the rats were acclimated for one week under a 12-h light/12-h dark cycle (8:00 AM-8:00 PM light, 8:00 PM–8:00 AM dark). Then, the Jet lag group underwent a progressive disruption of the normal day-night timing as depicted in Fig. 1A, while the LD12:12 group continued to be kept under a 12-h light/12-h dark environment. After 8 weeks, 5 rats of each group were sacrificed every 4 h from 8:00AM to 8:00 AM the next day with a total of 7 time points (ZT0, ZT4, ZT8, ZT12, ZT16, ZT20, and ZT24) to observe the changes of core clock genes in the mandibular condylar cartilage of rats over a 24-h period. RNA was extracted from the cartilage tissue using the TRIzol method. The extracted RNA was reverse-transcribed into cDNA, and then the expression of core clock genes *Bmal1*, *Clock*, *Per1*, *Per2*, *Cry1*, and *Cry2* was measured using RT-qPCR. The specific animal allocation information can be found in Appendix Table 2 and Fig. 1

### Micro-CT analysis

5 condyles from different rats in each group were collected and fixed with 4% paraformaldehyde solution for 24 then scanned by Micro-CT (70 kV, 114 mA, 6.8 μm, Scanco.μCT 50). BS/TV, BV/TV, Tb.Th, Tb.Sp, and Tb.N were analyzed by μCT evaluation Program V6.6. Horizontal and sagittal cross-sectional three-dimensional reconstructions were performed for all samples, and then a cuboid area (1.5 × 0.5 × 0.5 mm) beneath the cartilage was selected for local reconstruction, allowing for three-dimensional observation of the morphology of trabecular bone beneath the cartilage.

### Histological analysis

After isolating the condyles of the rats, they were fixed in 4% paraformaldehyde for 24 h, followed by decalcification in 10% EDTA for 8 weeks with weekly changes of EDTA solution. The specimens were then dehydrated, embedded in paraffin, and sliced along the sagittal plane of the condyle, with each section having a thickness of 5 μm. H&E staining: deparaffinization of the sections was done using xylene, followed by hydration using graded alcohols. The sections were then stained with hematoxylin for 3 min, differentiated with 1% hydrochloric acid alcohol for 2 s, counterstained with eosin for 2 min, dehydrated with graded alcohols, cleared in xylene, mounted with neutral resin, and photographed under a microscope. Alcian Blue-Nuclear Fast Red staining: deparaffinization and hydration of the paraffin sections were done, followed by staining with Alcian blue solution for 5 min, rinsing with distilled water, counterstaining with nuclear fast red for 3 min, rinsing with distilled water, dehydration, cleared in xylene, and mounting. Safranin O-Fast Green staining: deparaffinization and hydration of the paraffin sections were done, staining with fast green solution for 5 min, rinsing with water for 2 s, and subsequent staining with safranin O for 20 s. The sections were then rapidly dehydrated in ethanol, cleared in xylene, and mounted. The thickness of the cartilage layer was measured on the H&E staining sections, and each specimen was scored using the modified Mankin scoring system.

### Immunohistochemistry

Paraffin sections were deparaffinized with xylene, hydrated with graded alcohols, rinsed with PBS, permeabilized with 0.1% Triton X-100, and then incubated with 3% hydrogen peroxide for 15 min to quench endogenous peroxidase activity. Antigen retrieval was performed by incubating the sections in 1 mM EDTA antigen retrieval solution (pH = 9) in a water bath at 95 ℃ for 45 min. After rinsing with PBS, the sections were blocked with 5% BSA at room temperature for 1 h. The sections were then incubated with primary antibodies MMP13 (1:600, ProteinTech, China), ADAMTS4 (1:100, Affinity, USA), ADAMTS5 (1:100, Affinity, USA), and β-CATENIN (1:1000, ProteinTech, China) at 4 ℃ overnight. After warming up at 37 ℃ for 30 min, the primary antibodies were washed off with PBS and the sections were incubated with a secondary antibody (1:400) at room temperature for 30 min. Following PBS rinsing, DAB staining was performed, and then the sections were counterstained with hematoxylin for 30 s. After rinsing with tap water, the sections were dehydrated with graded alcohols, cleared in xylene, and mounted with neutral resin. Three fields (anterior, middle, posterior fields of the condylar cartilage) were selected from each slide, and images were captured under a microscope. The average optical density was analyzed using Image J 1.52i (National Institutes of Health, Germany), and statistical analysis was performed using Prism 8.0 (GraphPad, USA).

### Western blot

After washing the cells with PBS, they were lysed on ice with RIPA lysis buffer for 30 min. The lysates were then centrifuged at 12,000 g at 4 °C for 20 min, and the proteins were denatured at 100 °C with loading buffer. And they were frozen in liquid nitrogen and then grinded into powder. After adding RIPA, the samples were lysed on ice for 40 min and then centrifuged at 12,000 g at 4 °C for 20 min. The supernatant was mixed with loading buffer and denatured at 100 °C. Electrophoresis was performed at a constant voltage of 80 V for 30 min followed by 120 V for 50 min. The proteins were transferred onto a PVDF membrane using a constant current of 200 mA for 100 min. The membrane was then blocked with 5% skim milk to block nonspecific binding. After washing with TBST for 3 × 5 min, the membrane was incubated overnight at 4 °C with primary antibodies MMP13 (1:1000, ProteinTech, China), ADAMTS4 (1:800, Affinity, USA), ADAMTS5 (1:800, Affinity, USA), β-CATENIN (1:2000, ProteinTech, China), GSK 3β (1:1000, Cell Signaling Technology, USA), and p-GSK 3β-Ser9 (1:1000, Cell Signaling Technology, USA). After washing with TBST for 3 × 5 min, the membrane was incubated with a secondary antibody (1:1000) at room temperature for 1 h. Following washing with TBST for 6 × 5 min, the membrane was exposed using an ECL substrate (Millipore, Germany) to visualize the target proteins. Quantitative analysis was performed using Image J.

### RT-qPCR

Total RNA was extracted from cultured chondrocytes or mandibular condylar cartilage using TRIzol (Invitrogen, USA) every 4 h and reverse transcribed to cDNA using PrimeScript™ RT Msater Mix (Takara, Japan). All the cDNA samples were subsequently conducted to polymerase chain reaction with Hieff UNICON® Universal Blue qPCR SYBR Green Master Mix (Yeasen, China). The expression of clock genes (*Per1*, *Per2*, *Clock*, *Cry1*, *Cry2*, *Bmal1*) and *Mmp13*, *Adamts4*, *Adamts5*, *β-Catenin* was calculated by using cycle threshold method (2^−△△Ct^). The primer sequence was attached in supplementary file. And the internal control gene was *Gapdh*.

### Isolation and culture of mandibular condylar chondrocytes

Mandibular condylar cartilage was isolated from the condyles of 12 male Sprague–Dawley rats (3–5 days old). After digestion with Type I collagenase and trypsin for 3 h, the cells were centrifuged and plated in high-glucose DMEM culture medium. Subsequent passages were performed when cells reached 90% confluence. Cells in passages 2 were used for further experiments.

### Down-regulation and up-regulation of *Per1* by lentivirus in mandibular condylar chondrocyte and cell treatment

Silencing or overexpression of *Per1* was achieved using lentiviral transduction. After 12 h of lentiviral transduction, the regular culture medium was replaced. Total protein and RNA samples were collected 24 h later. Nuclear and Cytoplasmic Protein Extraction Kit (Beyotime, China) was used to extracted the nuclear and cytoplasmic protein according to the protocol. Regular medium containing GSK3β/β-CATENIN pathway activator LiCl (20 mM) [26, 27] or inhibitor XAV-939 (1 μM) [28] were replaced after silencing or overexpression of *Per1* by lentivirus and total protein and RNA were collected 24 h later for western blot and RT-qPCR.

### Immunofluorescence staining for β-CATENIN nuclear translocation. After silencing and overexpression of *Per1*

Mandibular condylar chondrocytes were washed with PBS, fixed with 4% paraformaldehyde for 15 min, treated with 0.1% Triton-100 at room temperature for 5 min, and incubated with 5% BSA at room temperature for 1 h. The primary antibody against β-CATENIN (1:200, Proteintech, China) was incubated overnight at 4 °C. After washing with PBS, the cells were incubated with the secondary antibody (Alexa Flour 594, 1:400, Yeasen, China) at room temperature for 1 h. PBST was used for washing, and DAPI (1:1000, Beyotime, China) was added and incubated at room temperature for 5 min. After washing 5 times with PBST for 5 min each, the fluorescence was observed and imaged using a fluorescence microscope.

### Injection of adenovirus-associated virus for downregulation of *Per1* in the mandibular condylar cartilage in jet-lag-induced TMJOA rat model

**Fig. 2:**
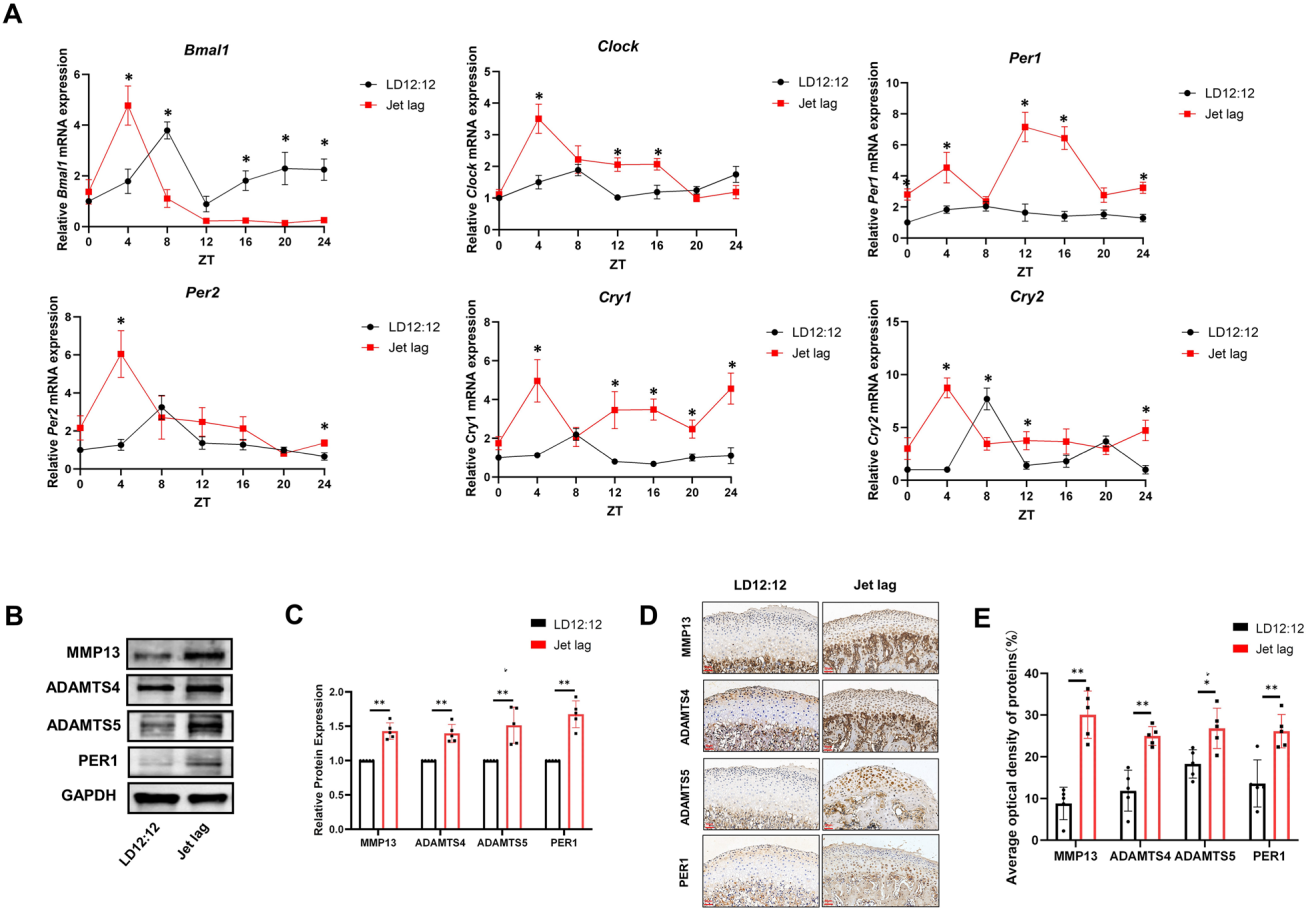
Chronic circadian rhythm disruption leads to increased expression of the clock gene *Per1* and matrix degrading enzymes in the mandibular condylar cartilage. **A** Relative mRNA expression curves of core clock genes in the mandibular condylar cartilage over a 24-h period (ZT0, ZT4, ZT8, ZT12, ZT16, ZT20 and ZT24) of two groups. **B** Western blot images of PER1, MMP13, ADAMTS4, and ADAMTS5 in mandibular condylar cartilage tissues in two groups. **C** Quantitative analysis of Western blot results. **D** Representative slices of immunohistochemical staining in the two groups. **E** Quantitative analysis of AOD in immunohistochemical staining of the two groups. n = 5, *P < 0.05. **P < 0.01

60 male Sprague Dawley rats (6w, 150–160 g) were selected and randomly divided into LD12:12 h + sh-Control, LD12:12 h + sh-*Per1*, Jet lag + sh-Control and Jet lag + sh-*Per1*groups (15 rats in each group). After one week of adaptation in a 12-h light/12-h dark environment, Jet lag + sh-Control and Jet lag + sh-*Per1*groups underwent jet lag using the aforementioned method. LD12:12 h + sh-Control and LD12:12 h + sh-*Per1* groups continued to be kept under a 12-h light/12-h dark environment. Adenovirus-associated viruses (OBiO Technology, China) corresponding to each group were injected into both TMJ cavities, with a volume of 10 µL per site. After 4 weeks, each rat was given a supplementary injection in each joint. After 8 weeks of modeling, the rats were sacrificed and the condyles were collected for Micro-CT scanning to measure bone parameters, immunohistochemistry, and tissue pathological staining. The condylar cartilage was isolated to extract tissue proteins for Western blot experiments. All animals were sacrificed at the same time of the day. The specific animal allocation information can be found in Appendix Table 3 and Fig. 2.

### Statistical analysis

All data were analyzed by Prism 8.0 (GraphPad, USA). In the animal experiment, the comparison between two groups of data was conducted using student* t*-test (as each group of animals is independent). The three biological replicates in the cell experiment were analyzed using paired *t*-test (with control and experimental groups set for each replicate). One-way ANOVA was used for comparisons across multiple groups, and Turkey’s test was used for post-hoc multiple comparisons. Prior to using* t*-test and one-way ANOVA, normality test and homogeneity of variance test were conducted on the data. Test level *α* = 0.05. All the quantitative data were presented as mean ± SD in figures.

## Results

### Chronic circadian rhythm disruption leads to a decrease in subchondral bone mass and TMJOA lesions in the rat mandibular condyles

As demonstrated by the three-dimensional reconstruction of the condyles in Fig. 1B, compared to the LD12:12 group, the Jet lag group showed a reduction in the volume of the mandibular condyle head, indicating absorption of the condyle. The three-dimensional reconstruction images of the trabeculae revealed that the trabeculae in the Jet lag group were sparser compared to those in the LD12:12 group. Quantitative analysis results shown in Fig. 1C indicate that the mandibular condyles of Jet lag group exhibited a significant decrease in BS/TV and BV/TV compared to the LD12:12 group, suggesting a significant reduction in subchondral bone mass in the Jet lag group compared to the LD12:12 group. Compared to the LD12:12 group, the Tb.Sp in the Jet lag group increased significantly, while the results of Tb.N decreased significantly, suggesting a reduction in trabeculae in the subchondral bone. These results all suggest that chronic circadian rhythm disruption can lead to the absorption of subchondral bone in the rat mandibular condyle. As shown in Fig. 1D with H&E staining results, the LD12:12 group displayed a normal number of chondrocytes, normal cellular arrangement, normal cartilage layer thickness, and dense subchondral trabeculae. In contrast, the Jet lag group exhibited blurred layers of cartilage cells, a significant reduction in the number of chondrocytes compared to the LD12:12 group, thinner cartilage, and sparse subchondral trabeculae. The results of Alcian Blue & Nuclear Fast Red and Safranin O & Fast Green staining in Fig. 1D showed that the LD12:12 group had intensely stained cartilage matrix that permeated the entire cartilage layer. In comparison, the cartilage matrix staining of the Jet lag group was noticeably lighter, suggesting a reduction in cartilage matrix due to chronic circadian rhythm disruption. In addition to the histological staining sections, quantitative analysis results of cartilage thickness in Fig. 1E also demonstrate a significant reduction in the thickness of the mandibular condylar cartilage layer in the Jet lag group compared to the LD12:12 group. As presented in Fig. 1F, the modified Mankin scores for the Jet lag group were significantly higher than that in the LD12:12 group. Taken together, these results suggest that chronic circadian rhythm disruption can lead to subchondral bone resorption, cartilage degeneration, and other osteoarthritis-like pathological changes in the rat mandibular condyles.

### Chronic circadian rhythm disruption leads to increased expression of the clock gene *Per1* and matrix-degrading enzymes in the mandibular condylar cartilage

To further investigate the role of clock genes in the TMJOA lesions caused by chronic circadian rhythm disruption in rats, we collected rat mandibular condylar cartilage at 4-h intervals, extracted RNA from the mandibular cartilage, and performed RT-qPCR to measure the changes in mRNA level of core clock genes *Clock, Bmal1*, *Per1*, *Per2*, *Cry*1, and *Cry2* over a 24-h period. As shown in Fig. 2A, the mRNA expression level of clock gene *Per1* in the Jet lag group was higher at all time points compared to LD12:12 group, whereas the 24-h expression curves of clock genes *Clock*, *Bmal1*, *Per2*, *Cry1*, and *Cry2* in the Jet lag group showed phase shifts compared to the LD12:12 group. These results suggest that chronic disruption of circadian rhythms may lead to increased expression of *Per1*, and the TMJOA lesions caused by chronic rhythm disruption may be associated with the circadian clock gene *Per1*.To further verify whether the expression of the circadian clock gene *Per1* is increased in the mandibular condylar cartilage of rats with chronic circadian rhythm disruption, we measured the expression of PER1 in the cartilage tissue protein of both the Jet lag and LD12:12 groups using Western blot and immunohistochemical staining. The results of immunohistochemical staining and Western blot results shown in Fig. 2B–E indicated that the expression of PER1 in the mandibular condylar cartilage of the Jet lag group was significantly higher than that of the LD12:12 group, suggesting that chronic circadian rhythm disruption can lead to increased expression of PER1 in rat mandibular condylar cartilage. To explore the specific molecular mechanisms by which chronic circadian rhythm disruption causes osteoarthritis lesions in rat mandibular condylar cartilage, we examined the expression of matrix-degrading enzymes MMP13, ADAMTS4, and ADAMTS5. As illustrated by the results of Western blot and immunohistochemical staining depicted in Fig. 2B–E, the expression of MMP13, ADAMTS4, and ADAMTS5 in the condylar cartilage of the Jet lag group was significantly higher compared to the LD12:12 group. This finding confirms that chronic circadian rhythm disruption not only leads to an increase in the expression of the clock molecule PER1 but also causes an increase of matrix-degrading enzymes in the rat mandibular condylar cartilage. This, in turn, promotes the occurrence and development of TMJOA in rats. However, whether chronic circadian rhythm disruption can promote the expression of matrix-degrading enzymes MMP13, ADAMTS4, and ADAMTS5 through clock gene *Per1*, thereby mediating the onset of TMJOA, requires further in-depth research.

### *Per1* positively regulates the expression of matrix-degrading enzymes in mandibular condylar chondrocytes

**Fig. 3:**
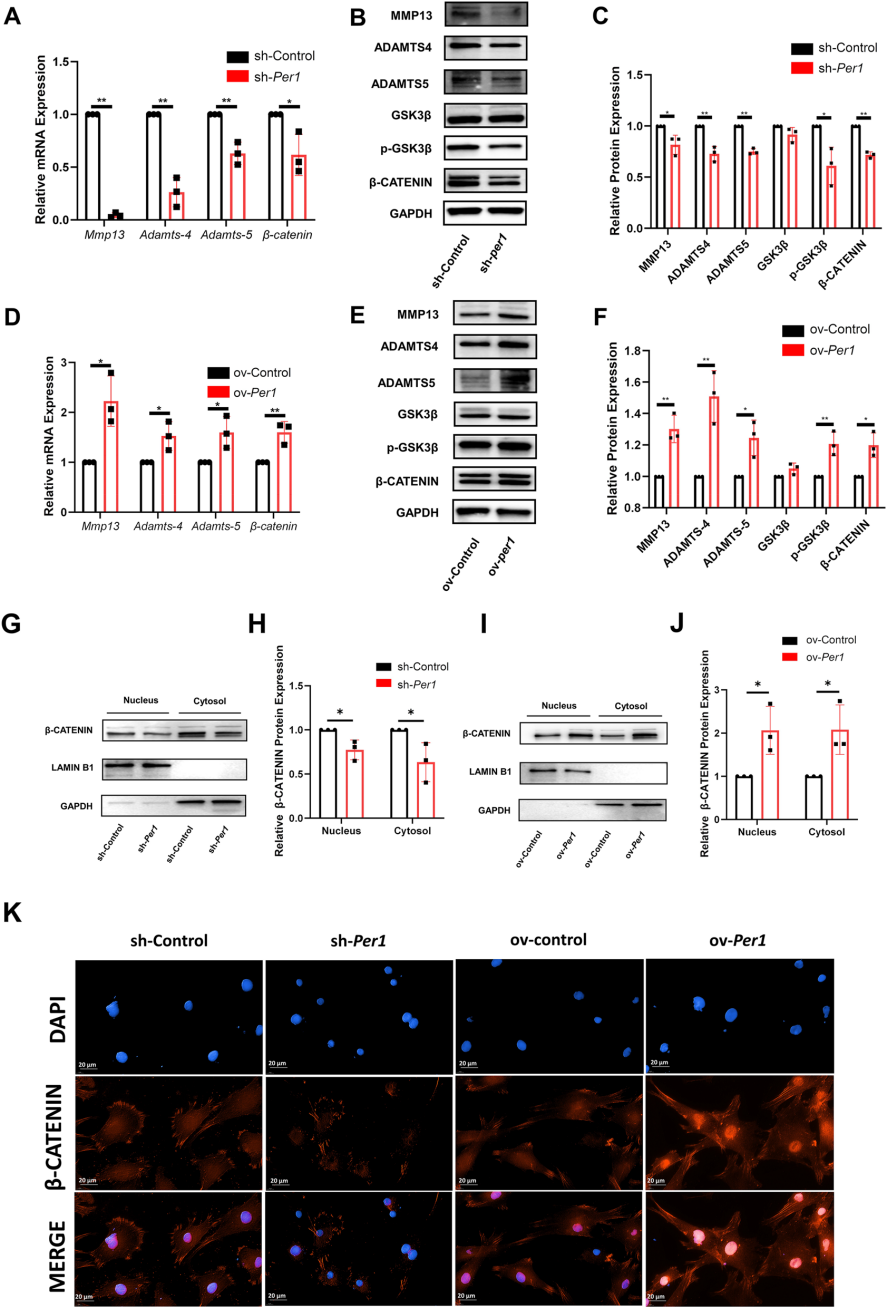
*Per1* positively regulates matrix-degrading enzymes and GSK3β/β-CATENIN pathway in mandibular condylar chondrocytes. **A** mRNA expression levels of *Mmp13*, *Adamts4*, *Adamts5* and core molecules of GSK3β/β-CATENIN pathway after downregulation of *Per1*. **B** Protein expression levels of MMP13, ADAMTS4, ADAMTS5 and core molecules of GSK3β/β-CATENIN pathway after downregulation of *Per1*. **C** Quantitative analysis of Western blot results after downregulation of *Per1*. **D** mRNA expression levels of *Mmp13*, *Adamts4*, *Adamts5* and core molecules of GSK3β/β-CATENIN pathway after upregulation of *Per1*. **E** Protein expression levels of MMP13, ADAMTS4, ADAMTS5 and core molecules of GSK3β/β-CATENIN pathway after upregulation of *Per1*. **F** Quantitative analysis of Western blot results after upregulation of *Per1*. **G** Protein expression levels of β-CATENIN in the nucleus and cytosol after downregulation of *Per1* in chondrocytes. **H** Quantitative analysis of Western blot results after downregulation of *Per1*. **I** Protein expression levels of β-CATENIN in the nucleus and cytosol after upregulation of *Per1* in chondrocytes. **J** Quantitative analysis of Western blot results after upregulation of *Per1*. **K** Immunofluorescence staining of β-CATENIN after downregulation or upregulation of *Per1* in mandibular condylar chondrocytes. n = 3, *P < 0.05. **P < 0.01

In animal experiments, we selected clock gene *Per1* as our target gene for study. To further verify whether *Per1* can influence the occurrence of TMJOA by affecting the expression of matrix-degrading enzymes, we transfected rat mandibular condylar chondrocytes with Sh-*Per1* and ov-*Per1* lentiviruses. First, we found no difference in the expression of *Per1* between the NC and sh-Control groups when downregulating *Per1* and no difference in the expression of *Per1* between the NC and ov-Control groups when upregulating *Per1* (Appendix Fig. 4), so we divided subsequent experiments into the sh-Control and sh-*Per1* groups or ov-Control and ov-*Per1* groups. Subsequently, we measured the mRNA and protein expression levels of matrix-degrading enzymes *Mmp13*, *Adamts4*, and *Adamts5* using RT-qPCR and Western blot. As shown in Fig. 3A–C, the expression of *Mmp13*, *Adamts4*, and *Adamts5* decreased significantly after downregulation of *Per1* in mandibular condylar chondrocytes. Conversely, after upregulation of *Per1*, the expression of *Mmp13*, *Adamts4*, and *Adamts5* in mandibular condylar chondrocytes increased significantly (Fig. 3D–F). These results suggest that clock gene *Per1* can positively regulate the expression of matrix-degrading enzymes including *Mmp13*, *Adamts4*, and *Adamts5*.

### Clock gene *Per1* can activate the GSK3β/β-CATENIN pathway in mandibular condylar chondrocytes

The results above have already demonstrated that clock gene *Per1* can positively regulate the expression of the matrix-degrading enzymes including MMP13, ADMATS4, and ADAMTS5 in mandibular condylar chondrocytes. To further explore the mechanisms, based on literature review, we focused our attention on the GSK3β/β-CATENIN pathway. After downregulation of *Per1* in rat mandibular condylar chondrocytes was downregulated via lentivirus, the mRNA expression of *β-catenin* significantly decreased (Fig. 3, A-C). Moreover, after downregulation of *Per1*, the protein expression of both p-GSK3β and β-CATENIN significantly decreased, while the expression of GSK3β showed no significant change, but the ratio of p-GSK3β/GSK3β significantly decreased. A reduction of p-GSK3β/GSK3β and β-CATENIN typically indicates that the GSK3β/β-CATENIN pathway is inhibited. Therefore, the following results show that the GSK3β/β-CATENIN pathway is inhibited after downregulating *Per1* in rat mandibular condylar chondrocytes. At the same time, after upregulating the expression of *Per1*, the mRNA expression of *β-catenin* significantly increased. Western blot results showed that after upregulating *Per1*, the protein expression levels of the core molecules of the GSK3β/β-CATENIN pathway also significantly increased. The expression of GSK3β did not change significantly, but the ratio of p-GSK3β/GSK3β increased significantly (Fig. 3D–F). This suggests that upregulation of *Per1* in mandibular condylar chondrocytes can activate the GSK3β/β-CATENIN pathway. In summary, clock gene *Per1* can positively activate the GSK3β/β-CATENIN pathway in rat mandibular condylar chondrocytes.

### *Per1* promotes the nuclear translocation of β-CATENIN in rat mandibular condylar chondrocytes

β-CATENIN is a downstream molecule of the GSK3β/β-CATENIN pathway. Once the pathway is activated, β-CATENIN enters the nucleus to exert its effects, thereby regulating the expression of downstream target genes. To further verify whether clock gene *Per1* can promote the nuclear translocation of β-CATENIN in rat mandibular condylar chondrocytes to exert its effects, we extracted nuclear and cytoplasmic proteins from condylar chondrocytes after downregulation or upregulation of *Per1* and separately detected the protein expression levels of β-CATENIN in the nucleus and cytoplasm. As indicated by the results shown in Fig. 3G–-J, β-CATENIN expression in both the nucleus and cytoplasm significantly decreased after downregulation of *Per1* in condylar chondrocytes. Similarly, after upregulation of *Per1* in mandibular condylar chondrocytes, protein expression of β-CATENIN in both the nucleus and cytoplasm significantly increased. This suggests that *Per1* can not only promote the expression of β-CATENIN within the cytoplasm of mandibular condylar chondrocytes but also promote its nuclear translocation. To better ascertain whether *Per1* can activate the GSK3β/β-CATENIN pathway and promote the nuclear translocation of β-CATENIN, we performed immunofluorescence staining of β-CATENIN on rat mandibular condylar chondrocytes after downregulation or upregulation of *Per1* to directly observe the nuclear translocation of β-CATENIN. As shown in Fig. 3K, the nuclear fluorescence intensity of β-CATENIN in the condylar chondrocytes decreased after downregulation of *Per1* compared to the sh-Control group, indicating a reduction in nuclear translocation of β-CATENIN. Conversely, after upregulation of *Per1* in chondrocytes, compared to the ov-Control group, the nuclear fluorescence intensity of β-CATENIN in the ov-*Per1* group increased, indicating an increase in nuclear translocation of β-CATENIN. Combining the results of immunofluorescence and nuclear-cytoplasmic Western blot experiments suggests that *Per1* can promote the nuclear translocation of β-CATENIN, successfully activating the GSK3β/β-CATENIN signaling pathway.

### *Per1* promotes the expression of matrix-degrading enzymes via GSK3β/β-CATENIN pathway in vitro

**Fig. 4:**
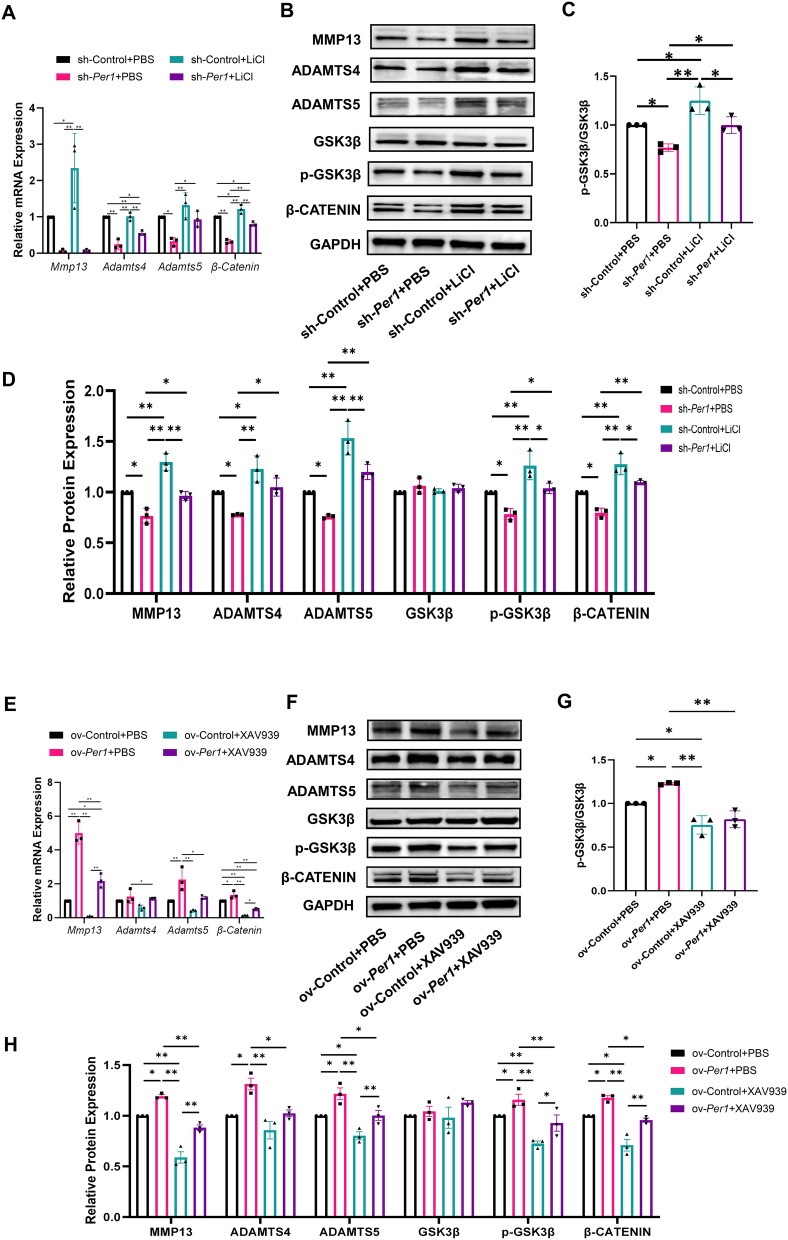
*Per1* regulates the synthesis of MMP13, ADAMTS4, and ADAMTS5 through GSK3β/β-CATENIN pathway in mandibular condylar chondrocytes. **A** mRNA expression levels of cartilage matrix degrading enzymes and *β-catenin* after downregulation of *Per1* in chondrocytes and addition of LiCl. **B** Protein expression levels of cartilage matrix degrading enzymes and core molecules in the GSK3β/β-CATENIN pathway after downregulation of *Per1* in chondrocytes and addition of LiCl. **C** Statistical analysis of p-GSK3β/GSK3β. **D** Statistical analysis of Western blot results. **E** mRNA expression levels of cartilage matrix degrading enzymes and *β-catenin* after upregulation of *Per1* in chondrocytes and addition of XAV939. **F** Protein expression levels of cartilage matrix degrading enzymes and core molecules in the GSK3β/β-CATENIN pathway after upregulation of *Per1* in chondrocytes and addition of XAV939. **G** Statistical analysis of p-GSK3β/GSK3β. **H** Statistical analysis of Western blot results. n = 3, *P < 0.05. **P < 0.01

To verify whether *Per1* promotes the synthesis of the matrix-degrading enzymes including MMP13, ADAMTS4, and ADAMTS5 in mandibular condylar chondrocytes by activating the GSK3β/β-CATENIN pathway, we added the LiCl (agonist of GSK3β/β-CATENIN pathway) and XAV939 (inhibitor of GSK3β/β-CATENIN pathway) to the culture medium of condylar chondrocytes after downregulation or upregulation of *Per1*. Afterwards, we assessed the expression of MMP13, ADAMTS4, ADAMTS5, and core molecules of the GSK3β/β-CATENIN pathway. As shown in Fig. 4A–D, downregulation of *Per1* in condylar chondrocytes suppressed the mRNA and protein expression of core molecules GSK3β/β-CATENIN pathway of and also inhibited the expression of MMP13, ADAMTS4 and ADAMTS5. However, LiCl rescued the inhibitory effects caused by downregulation of *Per1*, re-inducing the expression of matrix-degrading enzymes and re-activating the GSK3β/β-CATENIN pathway. As shown in Fig. 4E–H, upregulation of *Per1* in condylar chondrocytes increased the expression of core molecules GSK3β/β-CATENIN pathway of and MMP13, ADAMTS4 and ADAMTS5. XAV939 rescued the activating effects caused by upregulation of *Per1*, re-inhibiting the expression of matrix-degrading enzymes and the GSK3β/β-CATENIN pathway. Through bidirectional experimental validation, we confirmed that *Per1* can activate the GSK3β/β-CATENIN pathway in rat mandibular condylar chondrocytes, thereby promoting the expression of the matrix-degrading enzymes including MMP13, ADAMTS4, ADAMTS5, and consequently participating in the development and progression of TMJOA.

### Downregulation of *Per1* in rat mandibular condyle cartilage can ameliorate subchondral bone resorption and TMJOA lesions caused by chronic circadian rhythm disruption in rats

To validate the results of the cellular experiments at the animal level, we once again created a model of chronic circadian rhythm disruption in rats, and then injected sh-*Per1* adeno-associated virus into the temporomandibular joint cavity of the rats to downregulate the expression of *Per1* in mandibular condylar cartilage, observing its effect on the subchondral bone of mandibular condyles.

**Fig. 5:**
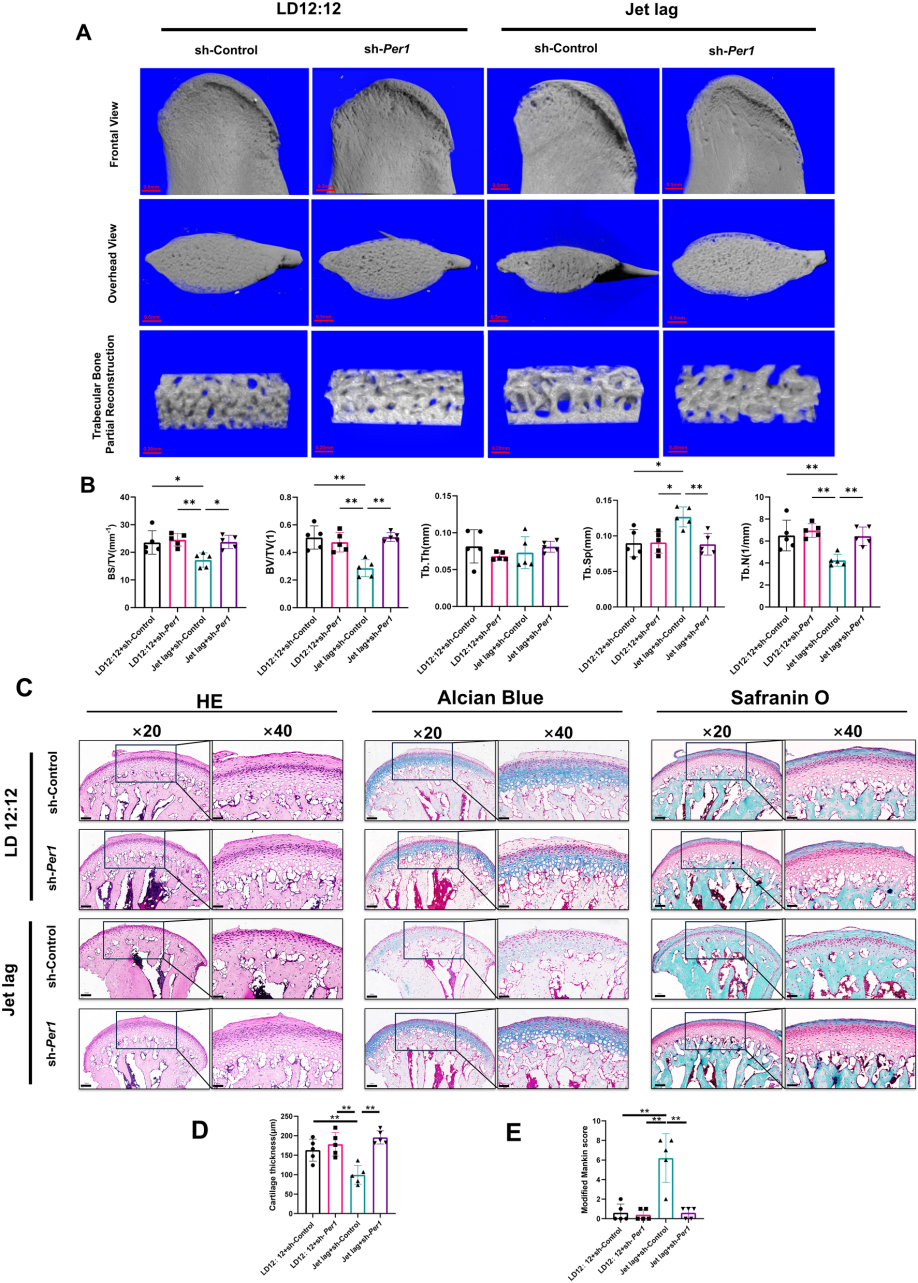
Downregulating *Per1* in rat mandibular condylar cartilage can alleviate TMJOA lesions caused by chronic circadian rhythm disruption in rats.** A** Three-dimensional reconstruction images of condyles from LD12:12 h + sh-Control, LD12:12 h + sh-*Per1*, Jet lag + sh-Control and Jet lag + sh-*Per1* groups by Micro-CT. **B** Quantitative evaluation of Micro-CT data including BS/TV, BV/TV, Tb.Th, Tb.Sp and Tb.N. **C** Representative sections of H&E, Alcian Blue-Nuclear Fast Red and Safranin O-Fast Green staining of four groups. **D** Cartilage thickness of four groups. **E** Modified Mankin scores of four groups. n = 5, *P < 0.05. **P < 0.01

As shown in the results of Micro-CT in Fig. 5A, there were no significant differences in the morphology of the mandibular condyles between the LD12:12 + sh-Control and LD12:12 + sh-*Per1* groups. Compared to the LD12:12 + sh-Control group, the condyle head in the Jet lag + sh-Control group showed obvious resorption, while in the Jet lag + sh-*Per1* group, the resorption of the condyle head was reduced, essentially returning to normal shape, proving that after the downregulation of *Per1* in the mandibular condylar cartilage, the resorption of condyles caused by chronic circadian rhythm disruption was improved. As shown in Fig. 5B, bony parameter analysis was consistent with morphological results, showing bone mass recovery in the Jet lag + sh-*Per1* group compared to the Jet lag + sh-Control group, resembling the normal group, which indicates that downregulation of *Per1* can ameliorate bony resorption caused by circadian rhythm disruption.

H&E, Alcian Blue & Nuclear Fast Red and Safranin O & Fast Green staining results revealed TMJOA-like lesions in the Jet lag + sh-Control group compared to the LD12:12 + sh-Control group, including disorganized cell layers, rough surface, and thinner cartilage. In the Jet lag + sh-*Per1* group, downregulation of *Per1* significantly improved the condition of the condylar cartilage, with cell layer organization, surface smoothness, and cartilage layer thickness approaching normal levels (Fig. 5C and D, indicating that downregulation of *Per1* effectively mitigates TMJOA lesions. Additionally, the results of modified Mankin score support this conclusion, with significantly lower scores in the Jet lag + sh-*Per1* group than in the Jet lag + sh-Control group (Fig. 5E), suggesting that downregulation of *Per1* has a positive effect on improving TMJOA lesions caused by chronic circadian rhythm disruption.

### Downregulation of *Per1* in rat mandibular condylar cartilage inhibits the GSK3β/β-CATENIN pathway and the increase of matrix-degrading enzymes caused by chronic circadian rhythm disruption

**Fig. 6:**
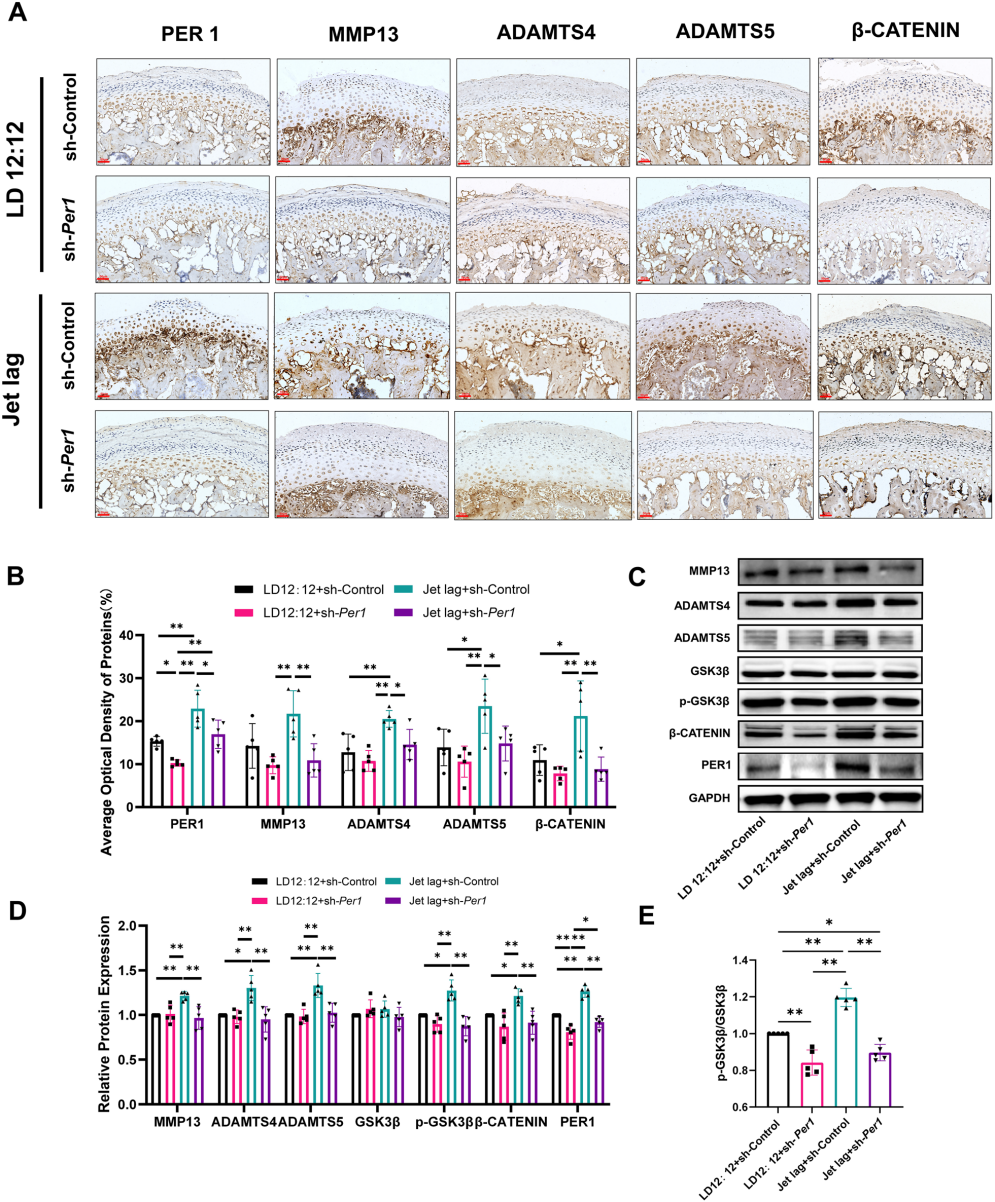
Downregulation of *Per1* in rat mandibular condylar cartilage can inhibit the activation of the GSK3β/β-CATENIN pathway and the increase in matrix-degrading enzymes caused by chronic circadian rhythm disruption. **A** Representative sections of immunohistochemical staining of PER1, MMP13, ADAMTS4, ADAMTS5 and β-CATENIN in mandibular condylar cartilage. **B** Average optical density of protein in condylar cartilage tissue. **C** Western blot results of PER1, MMP13, ADAMTS4, ADAMTS5, GSK3β, p-GSK3β, and β-CATENIN in the mandibular condylar cartilage of rats in four groups. **D** Statistical analysis of Western blot results in the condylar cartilage tissue of rats in the four groups. **E** Statistical analysis of p-GSK3β/GSK3β. n = 5, *P < 0.05, **P < 0.01

To further validate the molecular mechanisms, immunohistochemical staining was performed on four groups of condylar cartilages to examine changes in the expression of PER1, MMP13, ADAMTS4, ADAMTS5, and β-CATENIN. According to Fig. 6A and B, the expression of PER1 in the LD12:12 + sh-*Per1* group was significantly lower than in the LD12:12 + sh-Control group, and the expression of PER1 in the Jet lag + sh-*Per1* group was also significantly lower than in the Jet lag + sh-Control group. This proves that the sh-*Per1* adenovirus can effectively downregulate the expression of PER1 in the condylar cartilage. Moreover, the expression of β-CATENIN in the Jet lag + sh-Control group increased significantly compared to the LD12:12 + sh-Control group, further suggesting the activation of the GSK3β/β-CATENIN pathway during chronic circadian rhythm disruption. The expression of MMP13, ADAMTS4, ADAMTS5, and β-CATENIN in the Jet lag + sh-*Per1* group was significantly lower than in the Jet lag + sh-Control group, proving that downregulating *Per1* in condylar cartilage can reduce the increase in matrix-degrading enzymes expression and can inhibit the activation of the GSK3β/β-CATENIN pathway due to chronic circadian rhythm disruption. To further confirm these experimental results, proteins from the four groups of rat mandibular condylar cartilage tissues were extracted for Western blot experiments to detect the expression levels of PER1, MMP13, ADAMTS4, ADAMTS5, GSK3β, phosphorylated GSK3β (Ser9), and β-CATENIN. As shown in Fig. 6C–E, the expression of PER1 in the LD12:12 + sh-Per1 group was significantly lower than in the LD12:12 + sh-Control group, and the expression of PER1 in the Jet lag + sh-*Per1* group was also significantly lower than in the Jet lag + sh-Control group. This confirms the effective downregulation of PER1 expression in the condylar cartilage by the sh-*Per1* adenovirus, which is consistent with the results of the immunohistochemical staining mentioned above. Compared to the LD12:12 + sh-Control group, the expression of MMP13, ADAMTS4, ADAMTS5, p-GSK3β/GSK3β, and β-CATENIN in the Jet lag + sh-Control group were significantly higher, proving that chronic circadian rhythm disruption activates the GSK3β/β-CATENIN pathway and leads to an increase in the expression of matrix-degrading enzymes. This conclusion is consistent with previous findings. Compared to the Jet lag + sh-Control group, the expression of PER1, MMP13, ADAMTS4, ADAMTS5, p-GSK3β/GSK3β, and β-CATENIN in the Jet lag + sh-*Per1* group was significantly reduced, demonstrating that downregulation of *Per1* in rat condylar cartilage inhibits the activation of the GSK3β/β-CATENIN pathway and decreases the expression of matrix-degrading enzymes, thus mitigating the TMJOA-like lesions caused by chronic circadian rhythm disruption. This confirms our cellular experimental results in vivo.

## Discussion

Currently, there are limited researches on whether circadian rhythm disruption can lead to TMJOA. Our preliminary clinical research has found that chronotype affects the prevalence of TMDs, and there are differences in TMJ morphology among people with different chronotypes [29]. A study surveyed 663 college students, assessed their chronotype preference using the Morningness-Eveningness Questionnaire, and evaluated for TMDs using a self-assessment scale. They discovered that an evening chronotype is associated with the prevalence and severity of TMDs. Facial muscle pain and impaired lateral jaw movement scores were higher in evening and intermediate types, whereas morning type was a protective factor against TMDs [30]. These clinical studies indicate that circadian rhythm preference might impact the temporomandibular joint. However, these are cross-sectional studies that can only prove a correlation between circadian rhythm disruption and TMJ abnormalities, and it's still not clear whether circadian rhythm disruption is a risk factor causing TMJ abnormalities. Our study confirms that chronic circadian rhythm disruption is a risk factor for TMJOA, offering new insights into the prevention and treatment of TMJOA.

Multiple studies have shown that in nurses who do shift work, the expression of *PER1* in peripheral blood leukocytes is increased, suggesting that chronic circadian rhythm disruption may lead to an increase in the expression of *PER1* [31, 32]. However, currently there is limited research on the impact of chronic circadian rhythm disruption on clock genes in the mandibular condylar cartilage. Through jet lag rat model, we observed the TMJOA-like lesions in the rats of Jet lag group. By measuring the changes in the expression of core clock genes in both groups of mandibular condylar cartilage over a period of 24 h, we identified our target gene *Per1* in this study, which showed an increase in expression after circadian rhythm disruption. *Per1* is one of the core clock genes and acts as a negative regulator of the biological clock. When the clock molecule, BMAL1 and CLOCK dimer, initiates the synthesis of downstream genes, it also promotes the synthesis of negative regulators of biological clock like PER1. This, in turn, negatively regulates the synthesis of BMAL1 and CLOCK through a feedback loop, forming rhythmic oscillations [33]. PER1 is a transcription factor that directly regulates the transcription of downstream genes to exert specific functions [34]. There is also limited research on the role of PER1 in cartilage metabolism and differentiation. One study observed a 6-h phase difference in circadian rhythm between bone deposition and *Per1* expression in mouse skull, suggesting that *Per1* may regulate bone deposition in mouse skull [35]. Melatonin can promote the growth of mouse chondrocytes and the expression of cartilage differentiation-related genes *Col2a1*, *Acan,* and *Sox9*, inhibit the expression of type X collagen in chondrocytes, and melatonin can also inhibit the expression of clock gene *Per1*, enhancing the proliferative activity of chondrocytes. Biological clock genes play an important role in the proliferation and differentiation of chondrocytes [36]. Takarada et al. [37] discovered that rhythmic expression of *Per1* and *Ihh* in chondrocytes was disrupted after downregulation of *Bmal1*, leading to abnormal cartilage ossification in mice. Another study cultured mouse chondrocytes in a three-dimensional sponge and performed DNA microarray analysis, revealing the rhythmic expression of clock genes *Per1*, *Per2*, and *Clock* in chondrocytes, exhibiting a circadian rhythm [38]. After upregulation of *Per1* in the ATDC5 cells, the synthesis of cartilage matrix proteoglycan was significantly inhibited, and the mRNA expression of cartilage differentiation markers (including type II collagen and type X collagen) was significantly decreased. Following the isolation of chondrocytes from *Bmal1*^−/−^ mice, it was found that the rhythmic expression of clock gene *Per1* and cartilage differentiation-related factors was disrupted, and this disruption led to disorders in chondral ossification in the mice [37]. Our preliminary studies also found that rat mandibular condylar chondrocytes in vitro exhibited rhythmic expression of core clock genes *Per1*, *Per2*, *Clock*, *Cry1*, *Cry2*, *Bmal1*, and *Mmp13* under the influence of dexamethasone. The expression curve of *Mmp13* was most closely aligned with that of *Per1* in terms of phase and amplitude. Furthermore, under the stimulation of IL-1β, the expression of *Per1* in rat mandibular condylar chondrocytes increased. In a TMJOA rat model induced by unilateral anterior crossbite, the expression of PER1 in the condylar cartilage also significantly increased, suggesting that PER1 may play a certain role in the process of TMJOA induced by unilateral anterior crossbite [39]. Therefore, *Per1* likely also plays a regulatory role in the metabolism of mandibular condylar chondrocytes.

The mandibular condylar cartilage plays a crucial role in the onset and development of TMJOA. Injuries and degenerative changes of the condylar cartilage are usually the initiation of TMJOA. When there is erosion of the condylar cartilage, the temporomandibular joint lacks cushioning, and the occlusal force on the joint is directly exerted on the subchondral bone, leading to increased wear of the condyle. This results in condylar absorption and TMJ pain [9]. Clock genes can also regulate the synthesis of matrix-degrading enzymes and mediate the occurrence of osteoarthritis. After knocking out the clock gene *Bmal1* in chondrocytes, the expression of MMP13 significantly increased [40]. When chondrocytes were treated with IL-1β or hydrogen peroxide, the expression of BMAL1 decreases, the expression of SOX9 also decreased, and the expression of MMP13 and ADAMTS5 increased. Downregulation of *Bmal1* in chondrocytes promoted the expression of MMP13 and ADAMTS5, while downregulation of *Per2* inhibited the expression of MMP13 and ADAMTS5, demonstrating that arthritis and oxidative stress can promote the onset and development of osteoarthritis by affecting the expression of clock genes in chondrocytes [41]. Chronic circadian rhythm disruption could lead to osteoarthritis-like pathological changes in rat knees, with increased expression of MMP3, MMP13, and ADAMTS4 in knee cartilage and decreased expression of BMAL1. After downregulation of *Bmal1* in knee chondrocytes, the expression of MMP3, MMP13, and ADAMTS4 increased, proving that chronic circadian rhythm disruption can promote the synthesis of matrix-degrading enzymes by inhibiting the expression of *Bmal1*, thereby mediating the occurrence of knee osteoarthritis [42]. Sleep deprivation could activate the p-ERK pathway in rat mandibular condylar chondrocytes, increases the expression of matrix-degrading enzymes MMP1, MMP3, and MMP13, and leads to osteoarthritis-like lesions in the temporomandibular joint [43]. The above literature indicates that clock genes can participate in the pathogenesis of osteoarthritis by promoting or inhibiting the expression of matrix-degrading enzymes. Our cell experiments also showed that after downregulation of clock gene *Per1* in rat mandibular condylar chondrocytes, the expression of matrix-degrading enzymes MMP13, ADAMTS4, and ADAMTS5 decreased. Conversely, upregulation of *Per1* in mandibular condylar chondrocytes significantly increased the expression of MMP13, ADAMTS4, and ADAMTS5.

The GSK3β/β-CATENIN signaling pathway belongs to classic WNT pathway, which is one of the key pathways regulating cell proliferation, differentiation, and apoptosis. And it plays a significant role in maintaining the health and function of cartilage [44]. Abnormal activation of the GSK3β/β-CATENIN pathway is closely related to the enhanced degradation of cartilage matrix [45]. Increased expression of β-CATENIN has been found to directly increase the expression of MMP13, ADAMTS4, and ADAMTS5, ultimately leading to the degradation of joint cartilage matrix and the occurrence of osteoarthritis lesions [46, 47].

A study showed that adverse mechanical stress caused abnormal growth of the mandible and osteoarthritis-like lesions in the temporomandibular joint. The GSK3β/β-CATENIN pathway is activated in the mandibular condylar cartilage, leading to a significant increase in the expression of osteoarthritis-related cytokines. After applying mechanical force to mandibular condylar chondrocytes, the GSK3β/β-CATENIN pathway was activated, and the inhibitor of the GSK3β/β-CATENIN pathway, XAV939, could improve degenerative changes in cartilage caused by abnormal mechanical forces [28]. To confirm whether sustained activation of β-CATENIN in the middle and deep layers of the articular cartilage of the temporomandibular joint would disrupt the balance of the tissue, Wang et al. [48] constructed transgenic mice with specific activation of β-CATENIN, finding that the temporomandibular joints of mice gradually showed signs of cartilage degeneration, increased hypertrophic chondrocytes, thinning of cartilage, and a significant increase in the expression of *Mmp13* and *Adamts5* with age. To further investigate the role of *Mmp13* and *Adamts5* in TMJOA, transgenic mice with double gene mutations of *β-catenin*^(ex3)^/*Mmp13*^−/−^ and *β-catenin*^(ex3)^/*Adamts5*^−/−^ were constructed. It was observed that the degenerative changes in the condylar cartilage of the mice were significantly alleviated, suggesting that *β-catenin* is a key gene that triggers TMJOA, which can mediate osteoarthritis lesions by promoting the expression of *Mmp13* and *Adamts5*. Transgenic mice with specifically activated β-CATENIN in chondrocytes also showed weakened Alcian blue staining of the mandibular condylar cartilage, reduced number of superficial chondrocytes, increased number of deep hypertrophic chondrocytes, and severe morphological defects on the joint surface. This inhibited proliferation of condylar chondrocytes, promoted apoptosis, and significantly increased the expression of MMP13, COLX, ADAMTS4, and ADAMTS5 in the mandibular condylar cartilage [49]. The above literature suggests that the abnormal activation of the GSK3β/β-CATENIN signaling pathway is crucial in the development and progression of TMJOA. In this study, silencing or overexpression of *Per1* can inhibit or promote the expression of p-GSK3β, and β-CATENIN in mandibular condylar chondrocytes, thereby affecting the expression of MMP13, ADAMTS4, and ADAMTS5. Furthermore, activators and inhibitors of the GSK3β/β-CATENIN pathway can rescue these effects, which demonstrates that chronic circadian rhythm disruption can activate the GSK3β/β-CATENIN signaling pathway by upregulating the expression of *Per1* in rat mandibular condylar chondrocytes, promoting the expression of the matrix-degrading enzymes including MMP13, ADAMTS4, and ADAMTS5, and thereby mediating the occurrence of TMJOA.

**Fig. 7:**
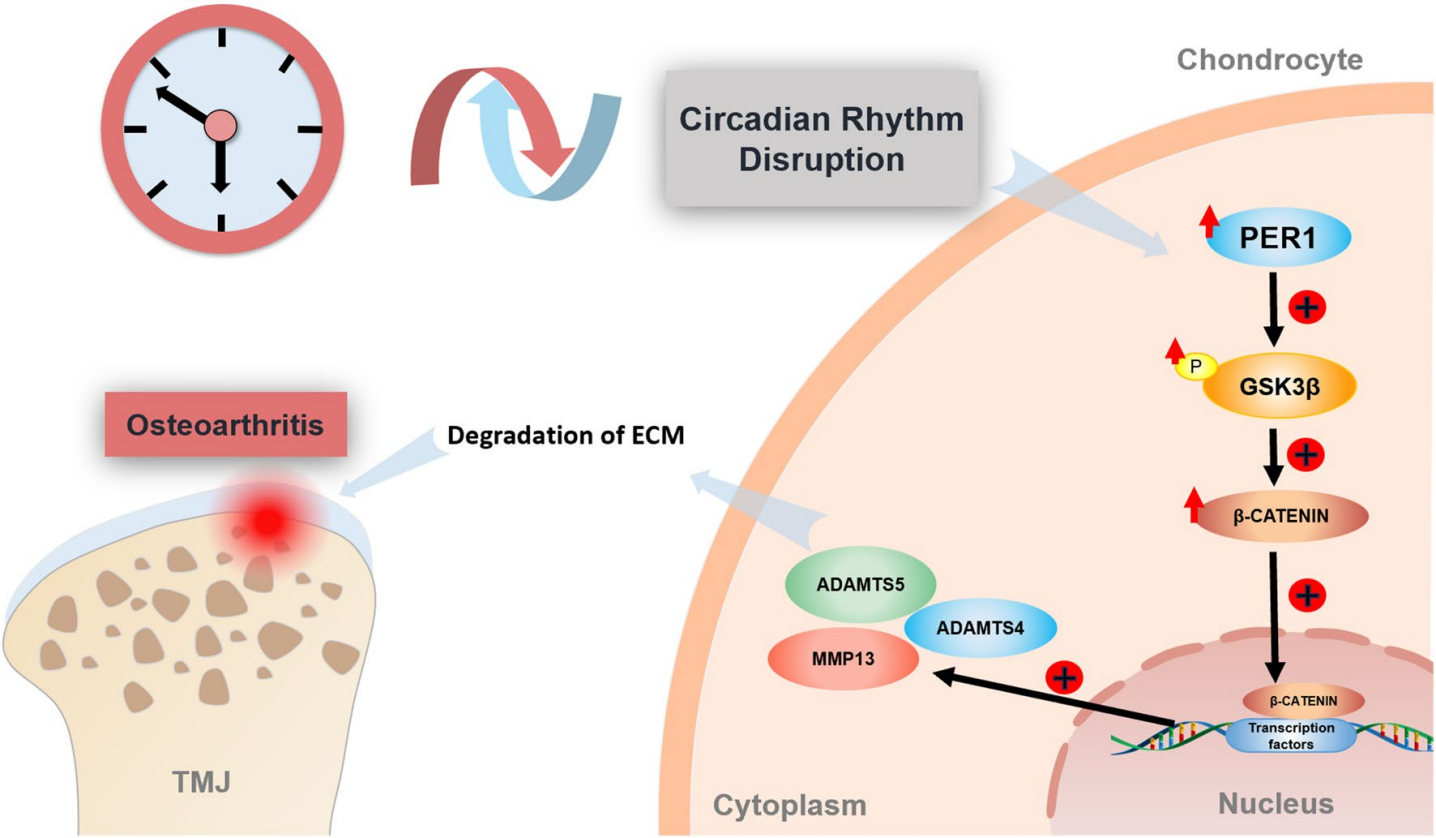
Schematic diagram of TMJOA mediated by upregulation of clock gene *Per1* due to chronic circadian rhythm disruption

In conclusion, this study shows that chronic disruption of circadian rhythms leads to increased expression of clock gene *Per1* in mandibular condylar chondrocytes, which in turn promotes the phosphorylation of GSK3β. This removes the inhibition of GSK3β on β-CATENIN, resulting in increasing nuclear translocation of β-CATENIN and enhancing synthesis of the downstream cartilage matrix-degrading enzymes including MMP13, ADAMTS4, and ADAMTS5, which leads to degradation of the cartilage matrix, degenerative changes, and ultimately mediates TMJOA lesions (Fig. 7).The findings of this study suggest that circadian rhythm plays a crucial role on the occurrence and development of TMJOA, and *Per1* may be a potential target gene for the treatment and prevention of TMJOA.

## Conclusions

Circadian rhythm disruption can lead to TMJOA. The clock gene *Per1* can promote the occurrence of TMJOA by activating the GSK3β/β-CATENIN pathway and promoting the expression of cartilage matrix-degrading enzymes. The clock gene *Per1* is a target for the prevention and treatment of TMJOA.

## Figures and Tables

**Fig. 8:**
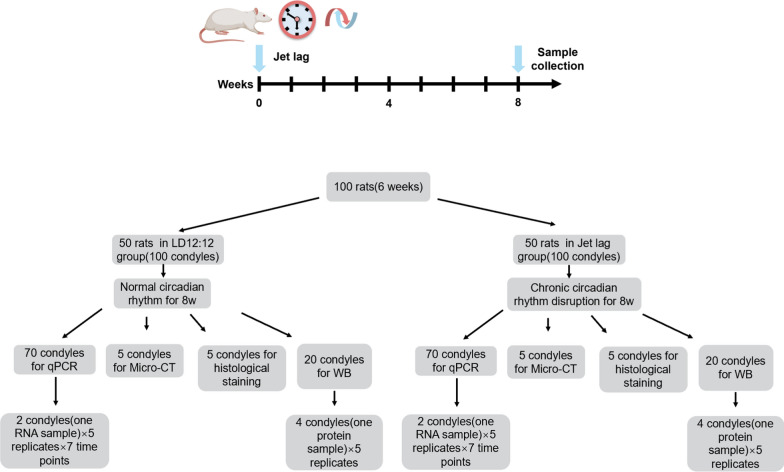
Schematic diagram of animal allocation for the first animal experiment

**Fig. 9:**
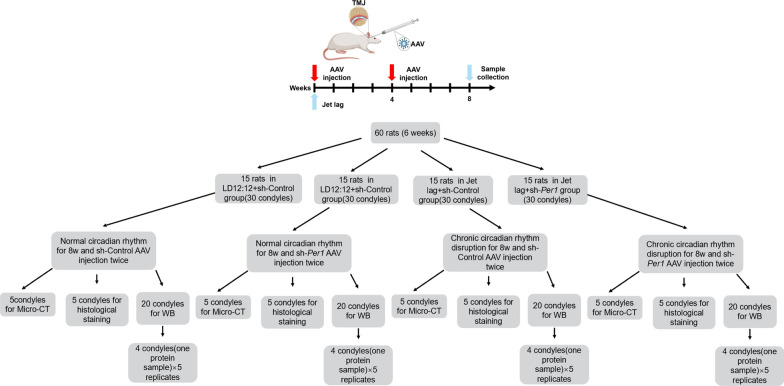
Schematic diagram of animal allocation for the second animal experiment

**Fig. 10:**
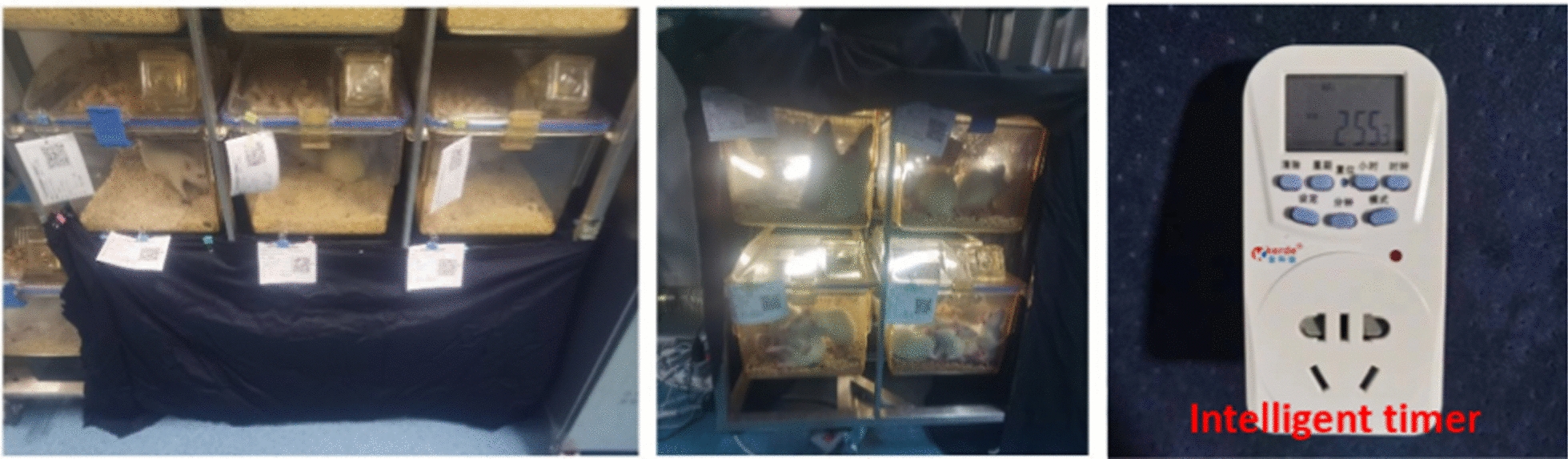
Modeling of chronic circadian rhythm disruption in rats using an independent lighting system controlled by an intelligent timer

**Fig. 11:**
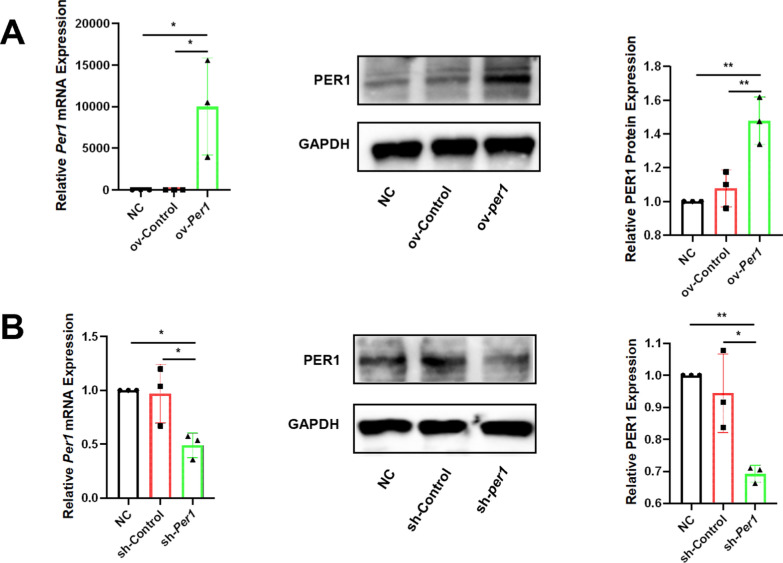
Verification of the efficiency of downregulation. **A** or upregulation **B** of *Per1* in mandibular condylar chondrocyte under sh-*Per1* and ov-*Per1* lentivirus. *P < 0.05. **P < 0.01

**Fig. 12:**
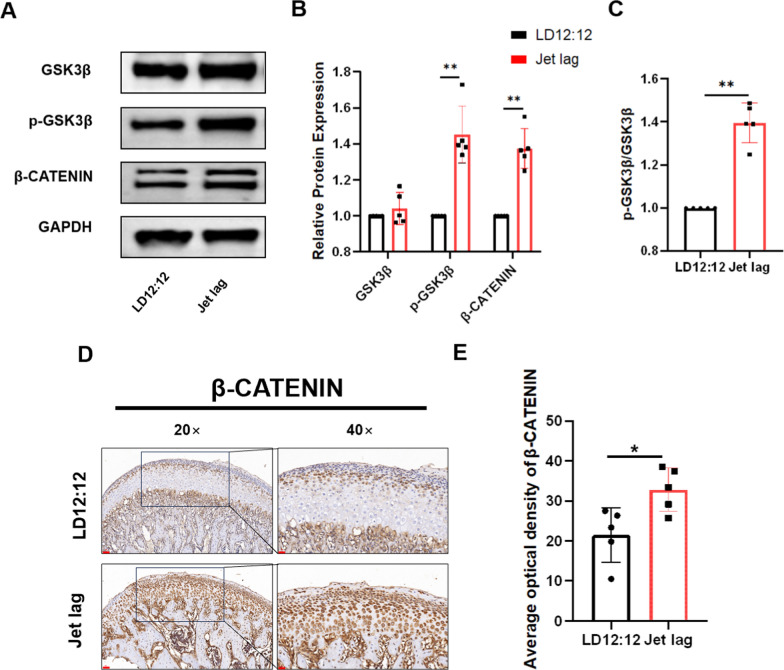
**A**, **B, C** Protein expression level of GSK3β, p-GSK3β and β-CATENIN in LD12:12 and Jet lag groups (n = 5). **D**, **E** Representative sections of immunohistochemical staining and average optical density of β-CATENIN in condylar cartilage tissue (n = 5). *P < 0.05. **P < 0.01

## Data Availability

The data that support the findings of this study are available from the corresponding author, upon reasonable request.

## Appendix

See Tables 1, 2 and 3, Figs. 8, 9, 10, 11 and 12.

**Table 1:** Primer sequence of core clock genes and cartilage-degrading factors

Gene	Sequence
*Mmp13*	Forward: TGCATACGAGCATCCATCCC Reverse: CGTGTCCTCAAAGTGAACCGC
*Adamts4*	Forward: GAAGAGTCCTGCCAGCAACC Reverse: ATGACCGTCAGCAGGTAGTG
*Adamts5*	Forward: TGCCCACCTAACGGCAAATC Reverse: GGACACCTGCGTATTTGGGA
*β-Catenin*	Forward: TGCGGCTGCTGTTCTATTCC Reverse: CCATCAACTGGATAGTCAGCACC
*Bmal1*	Forward: GAGGCGTCGGGACAAAATGA Reverse: GCTTCTGTGTATGGGTTGGTGG
*Clock*	Forward: ATCGGCAGCAAGAAGAACTAAG Reverse: TCAGTCCAGGGTTTGATTGCT
*Per1*	Forward: ACATCTGAATACACTCTCCGCAAC Reverse: GCAGGCGAGATGGTGTAGTAGAG
*Per2*	Forward: CCCAGCAAGTGATCGAGGACTA Reverse: TTGACACGCTTGGACTTCAGTT
*Cry1*	Forward: GTCCGACGACCATGATGAGAA Reverse: GCTTGCGAGCAGGGAGTTT
*Cry2*	Forward: ATGTGTTCCCAAGGCTTTTCAA Reverse: TGTAGGTAAGGGGTGGTTTCTGC
*Gapdh*	Forward: CTGGAGAAACCTGCCAAGTATG Reverse: GGTGGAAGAATGGGAGTTGCT

**Table 2:** The serial number of rat and distribution of mandibular condyles in LD12:12 and Jet lag groups

Micro-CT	IHC and histological staining	RT-qPCR	Western blot
Left condyles from #1–#5	Right condyles from #1–#5	Bilateral condyles from #6–#40 (The bilateral condylar cartilage of the same rat is pooled into one RNA sample. There are a total of 7 time points from ZT0 to ZT24, with 5 independent samples collected at each time point.)	Bilateral condyles from #41–#50 (Four pieces of mandibular condylar cartilage from two different rats were pooled into one protein sample, and five independent protein samples were collected from each group.)

Each group of rats was randomly assigned numbers #1 to #50

**Table 3:** The serial number of and distribution of mandibular condyles in LD12:12 h + sh-Control, LD12:12 h + sh-*Per1*, Jet lag + sh-Control and Jet lag + sh-*Per1* groups

Micro-CT	IHC and histological staining	Western blot
Left condyles from #1–#5	Right condyles from #1–#5	Bilateral condyles from #6–#15 (The bilateral mandibular condylar cartilage from 2 rats were pooled into one protein sample, and 5 independent protein samples were collected from each group.)

Each group of rats was randomly assigned numbers #1–#15
